# P-650. Cost-Effectiveness Analysis of Clesrovimab for Protection against Infant Respiratory Syncytial Virus Disease in the United States

**DOI:** 10.1093/ofid/ofaf695.863

**Published:** 2026-01-11

**Authors:** Klodeta Kura, John C Lang, Dawei Wang, Yoonyoung Choi, Michelle Goveia, Anushua Sinha, Yao-Hsuan Chen, Elamin Elbasha

**Affiliations:** MSD, London, England, United Kingdom; Merck Canada Inc., Kirkland, QC, Canada, Kirkland, Quebec, Canada; Merck Sharp & Dohme LLC, West Point PA, Pennsylvania; Merck, Philadelphia, PA; Merck, Philadelphia, PA; Merck & Co., Inc., Rahway, NJ, USA, Rahway, NJ; MSD, London, England, United Kingdom; Merck & Co., Inc., Rahway, New Jersey

## Abstract

**Background:**

Respiratory syncytial virus (RSV) causes significant hospitalization burden. Clesrovimab is an investigational, long-acting monoclonal antibody in development for the prevention of RSV lower respiratory tract infection in infants. The objective of this study was to evaluate the cost-effectiveness of introducing clesrovimab in US infants born during or entering their first RSV season.

**Figure 1:**
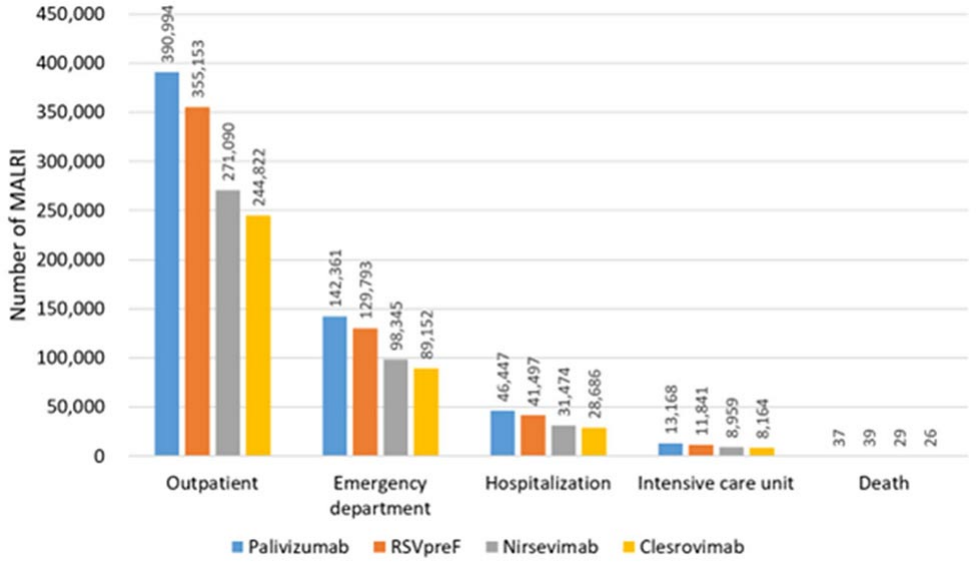
Number of MALRI estimated for (blue) palivizumab, (orange) RSVpreF, (grey) nirsevimab, and (yellow) clesrovimab.

**Figure 2:**
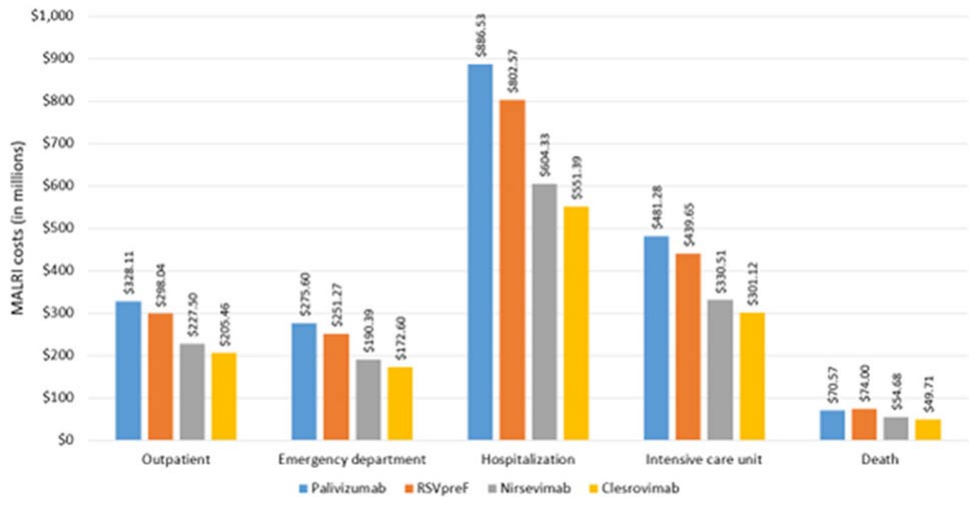
Economic impact of (blue) palivizumab, (orange) RSVpreF, (grey) nirsevimab, and (yellow) clesrovimab.

**Methods:**

A decision tree model simulated the clinical and economic impact of clesrovimab in infants, compared with three alternative interventions: nirsevimab, palivizumab, and RSVpreF vaccine. Model inputs were obtained from published literature. Efficacy estimate for clesrovimab resulted from a post-hoc analysis of the trial data, which was conducted to align endpoints from different studies. Health outcomes and costs (in 2024 USD) were evaluated from a societal perspective. Both deterministic and probabilistic sensitivity analyses were performed. Various efficacy assumptions were examined as scenario analyses.

**Results:**

Figures 1 and 2 illustrate the clinical and economic impacts of palivizumab, RSVpreF, nirsevimab, and clesrovimab. Clesrovimab resulted in fewer RSV-related medically attended lower respiratory infection (MALRI) outcomes and was cost-saving compared to nirsevimab, with significant reductions in total MALRI costs ranging from $33 million to $161 million.

When compared to palivizumab and RSVpreF, clesrovimab was estimated to cost between $7,372–$42,691 and $79,366–$134,353 per quality-adjusted life year (QALY), respectively. Results were sensitive to changes regarding intervention cost-per-dose parameters, efficacy assumptions, and QALY loss due to RSV infection.

**Conclusion:**

Based on listed parameters a single dose of clesrovimab can significantly reduce the burden of RSV among US infants and lead to cost-savings compared to nirsevimab.

**Disclosures:**

Klodeta Kura, PhD, MSD: Stocks/Bonds (Private Company) John C. Lang, PhD, MSc, MSc, BSc, Merck Canada Inc.: Stocks/Bonds (Private Company) Dawei Wang, PhD, Merck: Stocks/Bonds (Private Company) Yoonyoung Choi, PhD, MS, RPh, Merck & Co., Inc.: Grant/Research Support|Merck & Co., Inc.: Employment|Merck & Co., Inc.: Stocks/Bonds (Private Company) Michelle Goveia, MD, Merck: Stocks/Bonds (Private Company) Anushua Sinha, MD, Merck & Co., Inc., Rahway, NJ, USA: Employment|Merck & Co., Inc., Rahway, NJ, USA: Stocks/Bonds (Public Company) Yao-Hsuan Chen, PhD, MSD: Stocks/Bonds (Private Company) Elamin Elbasha, Ph.D., Merck & Co., Inc.: Employee|Merck & Co., Inc.: Stocks/Bonds (Public Company)

